# Lu^3+^ Substituted Gd_3_Ga_2_Al_3_O_12_:Ce Ceramics for Improved X-Ray Imaging

**DOI:** 10.3390/ma19173574

**Published:** 2026-08-23

**Authors:** Yuetong Zhen, Hui Lin, Yang Tang, Junwei Zhang, Yuchong Ding, Qiang Wang, Dawei Zhang, Jianren Xu

**Affiliations:** 1 Engineering Research Center of Optical Instrument and System, Ministry of Education and Shanghai Key Lab of Modern Optical System, University of Shanghai for Science and Technology, No. 516 Jungong Road, Shanghai 200093, China; 15261743396@163.com (Y.Z.);; 2 Research & Development Center of Materials and Equipment, China Electronics Technology Group Corporation No. 26 Research Institute, Chongqing 400060, China

**Keywords:** ceramic scintillator, scintillation decay time, afterglow, resolution of X-ray imaging

## Abstract

Ce^3+^-activated Gd_3_(Al,Ga)_5_O_12_:Ce scintillation ceramics have been widely studied due to their excellent scintillation properties. However, the relatively long radiative lifetime and slow decay components limit their applications in X-ray imaging. To address these issues, Lu^3+^ ions were introduced to partially substitute Gd^3+^ ions, thereby weakening the role of self-trapped states in the excitation process of Ce^3+^ and reducing the negative effects caused by shallow electron traps. As a result, the scintillation decay time was, overall, shortened, and an average decay time of 63 ns was obtained when x = 0.997. Meanwhile, the afterglow behavior induced by shallow electron traps was significantly suppressed (for the sample with x = 0.5, the afterglow intensity was measured to be approximately 0.48% of the initial intensity at 100 ms after the X-ray excitation was turned off). Meanwhile, an X-ray imaging spatial resolution comparable to that of commercial CsI:Tl (10 lp mm^−1^) was achieved for the (Gd,Lu)_3_Ga_2_Al_3_O_12_:Ce^3+^ scintillation ceramics.

## 1. Introduction

X-ray scintillators can absorb X-ray photons and convert them into ultraviolet or visible photons, and have been widely used in industrial non-destructive testing, medical imaging, X-ray computed tomography (X-CT), and high-energy physics [1,2,3,4]. For both static and dynamic X-ray imaging, scintillators play a critical role in determining image quality, including spatial resolution, contrast, signal-to-noise ratio, detective quantum efficiency, and temporal response [5,6,7,8,9,10,11]. Therefore, scintillation materials with high X-ray absorption efficiency, high light yield, fast decay, excellent stability, and strong radiation resistance are highly desired. At present, commonly used commercial X-ray scintillators include CsI:Tl, Gd_2_O_2_S:Tb (GOS), CdWO_4_, and (Lu,Y)SiO_5_ (LYSO). To provide a clearer comparison of their advantages and limitations in X-ray imaging applications, Table 1 summarizes the key optical and scintillation properties of these representative scintillators and the materials developed in this work. As shown in Table 1, although conventional commercial scintillators are widely used, their applications are still limited by long afterglow, insufficient light output, rigid bulk structures, or complicated fabrication processes [12,13,14]. In recent years, Ce^3+^-activated rare-earth garnet ceramics have attracted considerable attention due to their high light yield, high density, good energy resolution, and non-hygroscopic nature [15,16,17]. Among them, GAGG:Ce (Gd_3_Ga_2_Al_3_O_12_:Ce) is regarded as a promising scintillator because of its high light yield, good energy resolution, radiation tolerance, and high density [18,19]. However, its relatively long decay time and slow decay components still restrict its application in high-resolution X-ray imaging. Kitaura et al. found that, in GAGG:Ce, Gd^3+^ tends to occupy incorrect lattice sites and form anti-site defects, which can further combine with oxygen vacancies to form defect complexes [20]. These defects act as shallow electron traps, delaying carrier release and enhancing afterglow. Ionic substitution has therefore been explored to regulate the crystal structure and defect distribution. For example, Y^3+^ substitution can accelerate scintillation decay, although slow components remain in (Gd,Y)_3_Ga_2_Al_3_O_12_:Ce (GYGAG:Ce) ceramics due to defects and Ce^3+^ self-absorption [21,22]. Therefore, exploration of new co-dopant ions remains necessary for further improvement in the X-ray imaging performance of GAGG:Ce. According to previous reports [23]. In addition, optimization of the raw-material preparation process can reduce Ga_2_O_3_ volatilization during synthesis [24]. Moreover, replacing Gd^3+^ with Lu^3+^ can increase the effective atomic number and X-ray attenuation capability, while shortening the scintillation decay time and suppressing afterglow [25]. Therefore, Lu^3+^ doping is considered to be an effective strategy to improve the scintillation performance of GAGG:Ce.

In this work, a series of (Lu,Gd)_3_Ga_2_Al_3_O_12_:Ce ceramics were prepared by solid-state reactive sintering in a pure oxygen atmosphere. The effects of Lu^3+^ substitution on phase structure, defect evolution, scintillation properties, and X-ray imaging performance were systematically investigated. The relationship between composition, trap behavior, and scintillation response was further clarified to provide guidance for the design of high-performance garnet ceramic scintillators.

## 2. Experimental

A series of (Gd_0.997−x_Lu_x_Ce_0.003_)_3_Ga_2_Al_3_O_12_ (x = 0.1, 0.5, 0.7 and 0.997) transparent ceramics were successfully fabricated via solid-state sintering using Gd_2_O_3_ (99.99%, Aladdin, Shanghai, China), Lu_2_O_3_ (99.99%, Aladdin, Shanghai, China), Ga_2_O_3_ (99.999%, Shanghai, Aladdin, Shanghai, China), Al_2_O_3_ (99.99%, Aladdin, Shanghai, China), and CeO_2_ (99.99%, Aladdin, Shanghai, China) as raw materials, with the addition of 0.1 wt% MgO and 0.5 wt% tetraethyl orthosilicate (TEOS) as the sintering aids, and 1.0 wt% polyethylene glycol (PEG-400) as a dispersant. The commercial GAGG:Ce single crystal scintillator, used as the reference material, was prepared using Gd_2_O_3_ (99.99%, Aladdin, Shanghai, China), Ga_2_O_3_ (99.99%, Aladdin, Shanghai, China), Al_2_O_3_ (99.99%, Aladdin, Shanghai, China), and CeO_2_ (99.99%, Aladdin, Shanghai, China) as raw materials. The 20 g raw materials were weighed according to the stoichiometric ratio and mixed with 20 mL of ethanol, followed by planetary ball milling for 12 h. Subsequently, the obtained slurry was dried in an oven at 100 °C for 10 h. The dried powder was sieved three times through a 100-mesh screen and then calcined in a muffle furnace at 800 °C for 5 h to remove organic residues. The calcined powder was sequentially shaped by uniaxial dry pressing at 25 MPa and cold isostatic pressing at 250 MPa to obtain dense green bodies. The green bodies were then sintered at 1700 °C for 5 h in a pure oxygen atmosphere, followed by natural furnace cooling without subsequent annealing. Finally, the sintered ceramics were cut and polished on both sides to a thickness of 0.5 mm for further characterization. During polishing, the samples were first ground on a grinding machine using W20 SiC abrasive for approximately 1 h per surface, followed by W7 abrasive for final grinding. The surfaces were then polished sequentially using a high-speed polishing machine with W1 polishing slurry for 6 h, and a variable-frequency low-speed polishing machine with W0.5, W0.25, and W0.125 polishing slurries for 2 h, 1 h, and 1.5 h, respectively.

X-ray diffraction (XRD) analysis was conducted on a diffractometer (Model MiniFlex 600, Rigaku, Tokyo, Japan) with Cu K_α_ radiation at 40 kV and 15 mA to examine the crystal structure and phase purity of the samples over a 2θ range of 10–90°. The microscopic morphology was characterized using a scanning electron microscope (JEOL JSM-1001F, Tokyo, Japan) coupled with an energy dispersive spectrometer (EDS). The Steady photoluminescence (PL), PL excitation (PLE) spectra, and luminescence decay curves were recorded by a fluorescence spectrophotometer (FLS-1000, Edinburgh Instruments, Edinburgh, UK). For scintillation characterization, X-ray excited luminescence (XEL) spectra were acquired using a fluorescence spectrometer (OmniFluo 900, Zolix, Beijing, China) equipped with a tungsten X-ray tube operating at 30 kV and 500 μA. The light yield was digitized and processed with a multi-channel analyzer (CR105, Hamamatsu, Hamamatsu, Japan). Scintillation decay profiles were recorded using a high-speed oscilloscope (THS3024, Tektronix, Beaverton, OR, USA). Prior to afterglow and thermoluminescence (TL) measurements, (Lu,Gd)_3_Ga_2_Al_3_O_12_:Ce (GLAGG:Ce) ceramic samples with different compositions were irradiated with X-ray for 10 min. The afterglow signals were monitored at room temperature using a spectrometer (Zolix, OmniFluo 900, China); TL curves were measured with a TL instrument (TOSL-3DS, Guangzhou Rongfan, Guangzhou, China) by heating the samples from 100 K to 600 K at a rate of 1 K/s.

Overall, the X-ray imaging system consists of a fluorescence spectrometer (Zolix, OmniFluo 900, China), a resolution test chart, the (Gd_0.497_Lu_0.5_Ce_0.003_)_3_Ga_2_Al_3_O_12_ ceramic sample, a capsule spring, a chip, and an optical camera.

## 3. Results and Discussions

**Figure 1:**
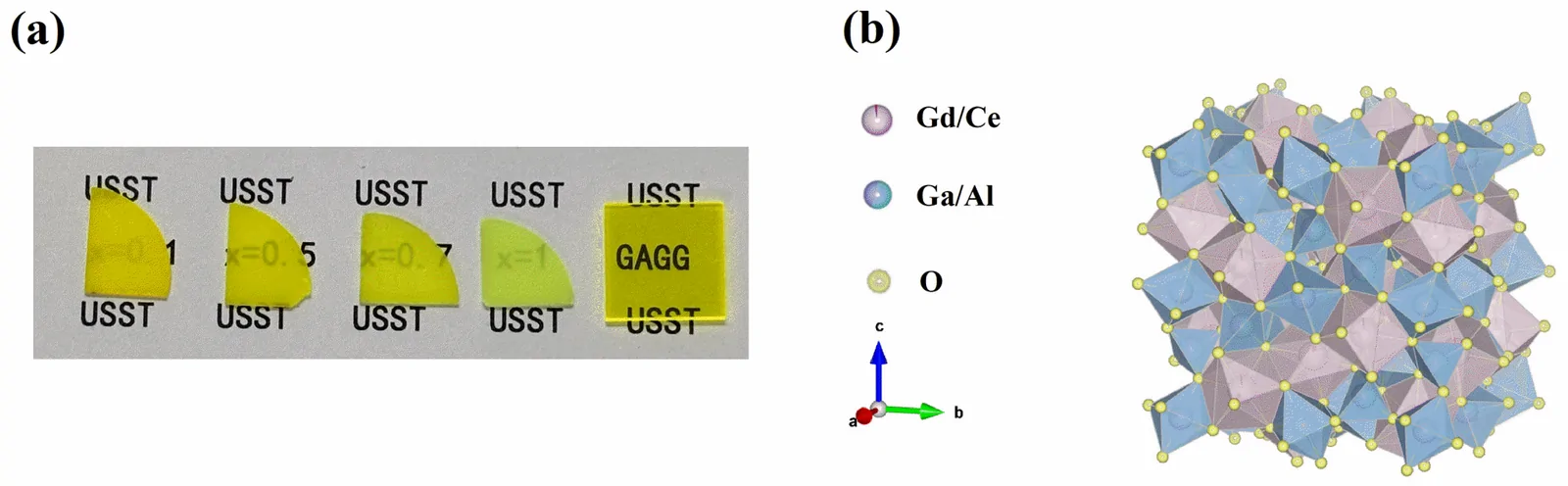
(**a**) The images of (Gd_0.997−x_,Lu_x_)_3_Ga_2_Al_3_O_12_:0.003Ce^3+^ (x = 0.1, 0.5, 0.7 and 0.997) ceramics and GAGG:Ce^3+^ crystal. (**b**) Crystal structure of (Gd,Lu)_3_Ga_2_Al_3_O_12_:Ce ceramics.

Figure 1a displays photographs of (Gd_0.997−x_Lu_x_Ce_0.003_)_3_Ga_2_Al_3_O_12_ (x = 0.1–0.997) scintillation ceramics under natural light. With increasing Lu^3+^ content, the sample color gradually changes from yellow to yellowish-green. Figure 1b illustrates the garnet crystal structure, which adopts a body-centered cubic framework with space group Ia$\overset{-}{3}d$, where [GaO_4_]/[AlO_4_] tetrahedra and [GaO_6_]/[AlO_6_] octahedra are interconnected via shared O^2−^ ions with distorted [GdO_8_] dodecahedra, forming a three-dimensional network [26]. The incorporation of smaller Lu^3+^ ions induces lattice contraction in the (Lu,Gd)_3_Ga_2_Al_3_O_12_ host [27].

**Figure 2:**
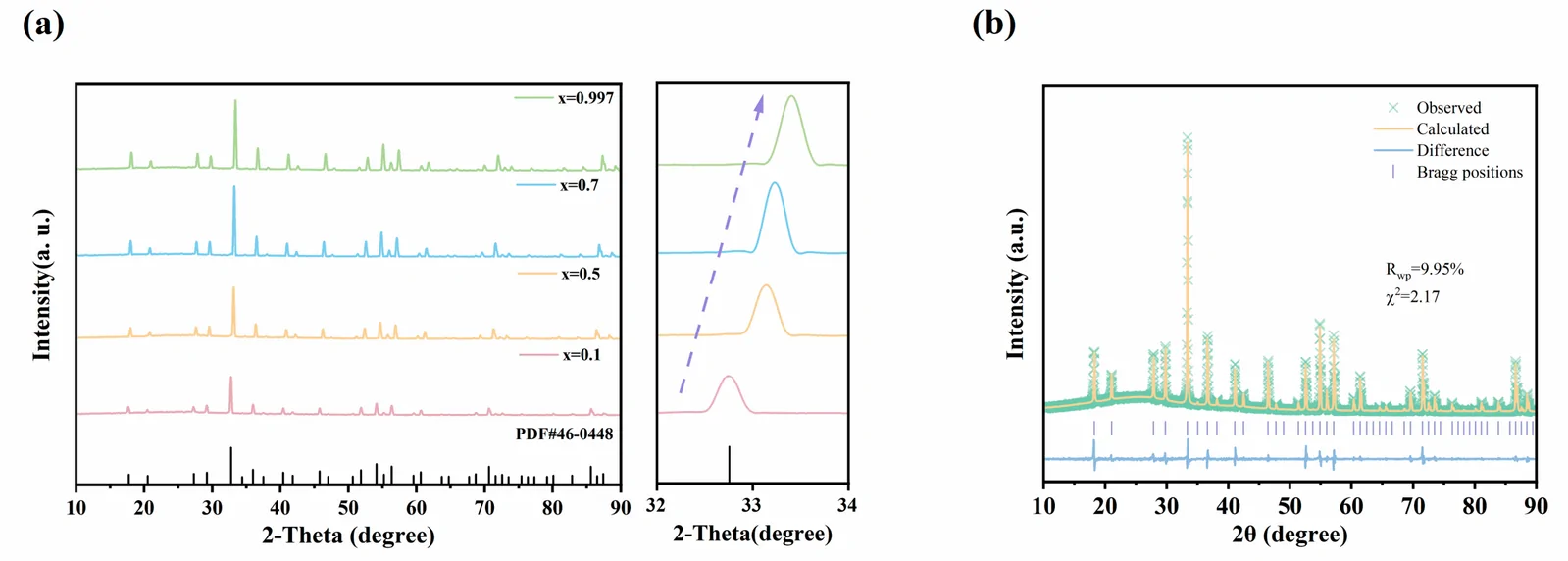
(**a**) XRD θ-2θ scans of (Gd_0.997−x_,Lu_x_)Ga_2_Al_3_O_12_:Ce^3+^ ceramics with different Lu^3+^ concentrations. (**b**) Rietveld refinement XRD pattern of (Gd_0.497_,Lu_0.5_,Ce_0.003_)Ga_2_Al_3_O_12_ ceramic sample.

Figure 2a presents the XRD θ–2θ scans of all the samples, where all diffraction peaks align with the standard card (PDF#46-0448) with no detectable impurity phases, confirming the formation of pure-phase (Lu,Gd)_3_Ga_2_Al_3_O_12_:Ce ceramics. The enlarged diffraction peak near 2θ = 33° shifts systematically toward higher angles with increasing Lu^3+^ content, indicating lattice contraction caused by the substitution of smaller Lu^3+^ ions for larger Gd^3+^ ions. Rietveld refinement was performed for the representative sample with x = 0.5 using the GSAS-II software package, based on the corresponding CIF structural file as the initial model. The refinement results show good agreement between the experimental and calculated patterns, with low Rwp and χ^2^ values of 9.95% and 2.17, respectively, as shown in Figure 2b.

**Figure 3:**
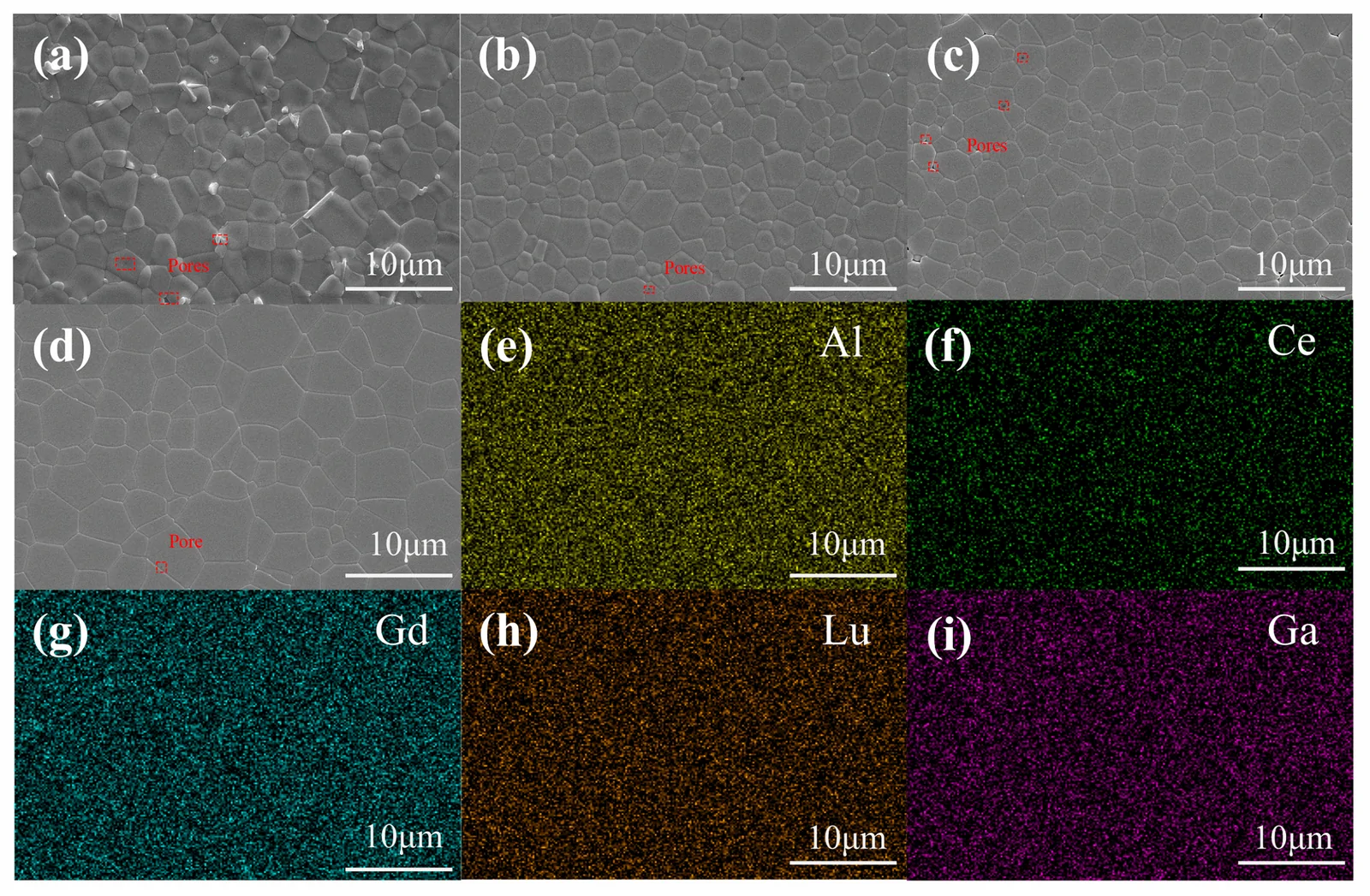
(**a**–**d**) SEM images of the (Gd_0.997−x_,Lu_x_)_3_Ga_2_Al_3_O_12_:0.003Ce^3+^ (x = 0.1, 0.5, 0.7 and 0.997) ceramic samples. (**e**–**i**) EDS surface mapping to disclose the elemental distributions of Al, Ce, Gd, Lu and Ga of the (Gd_0.497_,Lu_0.5_,Ce_0.003_)Ga_2_Al_3_O_12_ ceramic.

As shown in Figure 3a–d, the surface SEM morphology of (Gd_0.997−x_,Lu_x_)_3_Ga_2_Al_3_O_12_:0.003Ce^3+^ (x = 0.1, 0.5, 0.7 and 0.997) ceramic samples sintered at 1700 °C in an oxygen atmosphere was observed by SEM in secondary electron (SE) imaging mode, revealing dense microstructures with clear grain boundaries. A small number of residual pores were observed, which may be related to the high sintering temperature and the different diffusion rates of ions in the multicomponent solid-solution system. These pores can act as light-scattering centers during scintillation light propagation, which may adversely affect the spatial resolution of X-ray imaging. The elemental mapping images in Figure 3e–i reveal a uniform distribution of Al, Ce, Gd, Lu, and Ga in the x = 0.5 ceramic, indicating the formation of a homogeneous solid solution. Point EDS analysis further confirms that the elemental ratios are close to the designed composition, suggesting successful incorporation of Ce^3+^ and the constituent elements into the GLAGG garnet lattice, as shown in Figure S1 and Table S1. The optical transmission spectra of GLAGG:0.3at%Ce^3+^ ceramics with different Lu^3+^ doping concentrations are shown in Figure S2. The absorption band located at around 450 nm is assigned to the 4f–5d transition of Ce^3+^. The low overall transmittance of the samples is due to the light scattering caused by the remaining porosity and the secondary phases.

**Figure 4:**
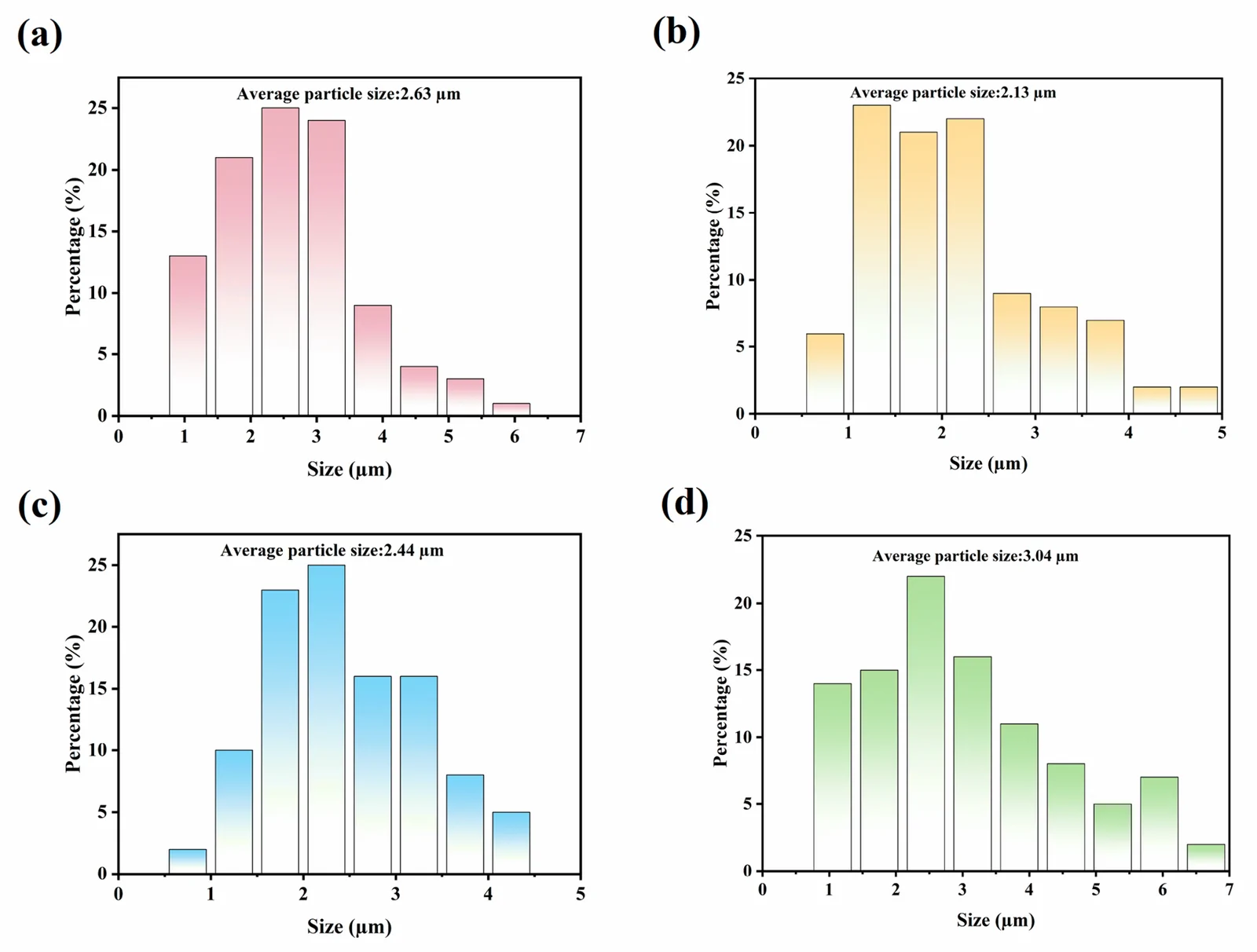
Grain-size distributions of (Gd_0.997−x_,Lu_x_)_3_Ga_2_Al_3_O_12_:0.003Ce^3+^ ceramics: (**a**) x = 0.1, (**b**) x = 0.5, (**c**) x = 0.7, and (**d**) x = 0.997.

Furthermore, the average grain sizes of the samples were quantitatively measured from the SEM images using Nano Measure 1.2.5 image analysis software, as shown in Figure 4a–d. With increasing Lu^3+^ content, the average grain size first decreases and then increases. At low Lu^3+^ concentrations, lattice contraction and local strain suppress grain growth. At high Lu^3+^ concentrations, Lu^3+^ becomes the dominant A-site cation, which enhances grain-boundary migration and leads to abnormal grain growth [28,29].

**Figure 5:**
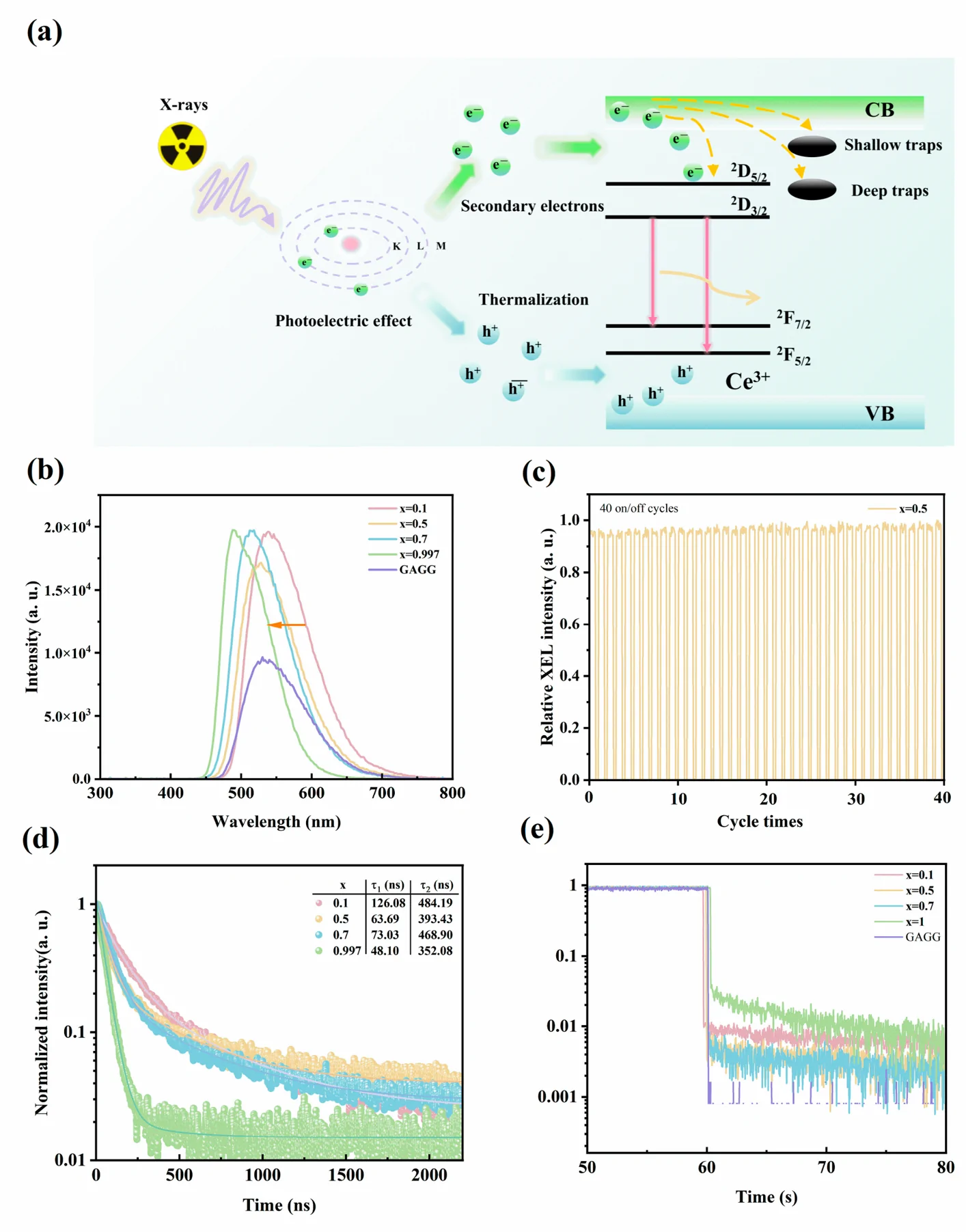
(**a**) Physical processes of scintillation. (**b**) XEL spectra of the Ce:GLAGG ceramic and the commercial GAGG:Ce single crystal reference. (**c**) X-ray switching cycles for 40 times of Ce:GLAGG. (**d**) The scintillation decay curves of Ce:GLAGG ceramics under ^137^Cs radiation (662 keV). (**e**) X-ray-induced afterglow of the Ce:GLAGG ceramic and the commercial GAGG:Ce single-crystal reference.

The XEL behavior of the samples is illustrated in Figure 5a. Under X-ray excitation, Ce^3+^-doped scintillators undergo energy deposition, carrier generation, migration, energy transfer, and radiative recombination rather than direct excitation of Ce^3+^ centers. The generated carriers transfer part of their energy to Ce^3+^ centers, leading to the 5d→4f radiative transition and characteristic yellow-green emission. Meanwhile, some carriers may be captured by shallow traps, deep traps, or Ce^4+^ recombination centers [30]. The XEL behavior spectra of (Gd_0.997−x_Lu_x_Ce_0.003_)_3_Ga_2_Al_3_O_12_ ceramic samples and GAGG:Ce crystal are shown in Figure 5b. Under identical measurement conditions, all ceramic samples exhibit higher emission intensities than the commercial GAGG:Ce crystal. A broad emission band centered at around 550 nm is observed for all samples, which is assigned to the Ce^3+^ 5d→4f transition. The slight shift in emission position is related to changes in the crystal-field environment around Ce^3+^ [31]. To evaluate operational stability, X-ray on-off cycling tests were performed for 40 cycles. As shown in Figure 5c, the XEL intensity remains nearly unchanged during repeated irradiation, demonstrating good luminescence stability and repeatability under X-ray excitation.

The scintillation decay curves of samples with different Lu^3+^ contents are shown in Figure 5d. The decay time was obtained by fitting the pulse peak. With increasing Lu^3+^ content, the average decay time decreases from 317 ns (x = 0.1) to 63 ns (x = 0.997) [32]. This shortened decay time can be attributed to Lu^3+^ incorporation, which facilitates carrier transfer from the excited state to the ground state of Ce^3+^ [33]. The slow component is mainly related to energy transfer from Lu^3+^ and Gd^3+^ to Ce^3+^ and delayed recombination through shallow traps, consistent with previous reports on Ce:GLAGG ceramics [34,35]. Figure 5e illustrates the radiation-induced afterglow decay curves of the Ce:GLAGG ceramic samples with different Lu^3+^ contents and the commercial GAGG:Ce crystal. Compared with the low afterglow level of the commercial GAGG:Ce crystal, approximately 0.09%@100 ms, the ceramic samples show higher residual luminescence, indicating that their afterglow performance still requires further optimization. Among the ceramic samples, the x = 0.5 composition exhibits the lowest afterglow intensity, suggesting that appropriate Lu^3+^ substitution can help suppress afterglow [36]. Charge carrier traps, especially those associated with ceramic grain boundaries, are considered a major origin of afterglow [37]. Excessive Lu^3+^ incorporation may increase structural disorder and enhance the involvement of self-trapped states in the Ce^3+^ excitation process, thereby increasing afterglow intensity [38]. The different residual signals of the x = 0.997 sample in scintillation decay and afterglow measurements can be attributed to their different carrier dynamics and time scales: scintillation decay mainly reflects fast Ce^3+^-related recombination and shallow-trap processes on the nanosecond scale, whereas afterglow arises from carrier release from deeper traps on the millisecond or longer time scale.

**Figure 6:**
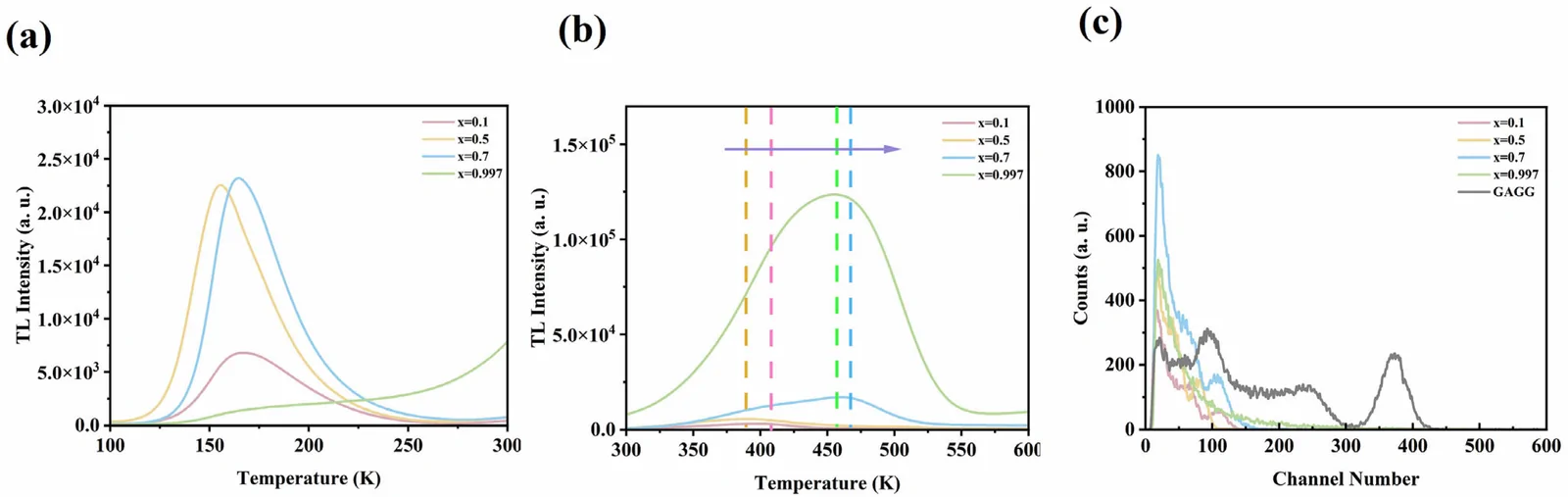
(**a**,**b**) Low-temperature and room-temperature TL glow curves of the sample measured after X-ray irradiation. (**c**) Pulse height spectra of samples under ^137^Cs radiation (662 keV).

Figure 6a,b show the TL glow curves of GLAGG ceramics after X-ray irradiation from 100 to 600 K. According to the trap-dynamics model of Ce-doped garnet scintillators, irradiation-generated carriers can be trapped and thermally released to recombine at Ce^3+^ centers. Thus, the TL peak position and intensity reflect trap depth and concentration, which are closely related to scintillation decay and afterglow behavior [30]. With increasing Lu^3+^ content, the low-temperature TL peak shifts toward lower temperature and becomes less distinct, indicating shallower traps and easier thermal release of trapped carriers. At excessive Lu^3+^ content, no obvious TL peak is observed, consistent with previous reports [20]. This behavior is associated with reduced Gd-related shallow traps and weakened excitation migration along the Gd sub-lattice after Lu^3+^ substitution [39,40]. Peaks above 300 K are generally assigned to deep traps. As shown in Figure 6b, the increased TL intensity with Lu^3+^ content suggests the formation of more deep traps, especially for the x = 0.997 sample, where excessive Lu^3+^ incorporation enhances local lattice distortion and cation disorder. These deep traps slowly release carriers after X-ray excitation is switched off, leading to stronger afterglow [41,42]. In contrast, the x = 0.5 sample exhibits lower TL intensity and a shift in the TL peak toward lower temperature, indicating a lower trap concentration and shallower trap depth. This suggests that appropriate Lu^3+^ substitution can suppress part of the Gd-related shallow traps without introducing abundant deep traps, allowing trapped carriers to be released more readily and recombine at Ce^3+^ luminescence centers [41,43]. However, excessive Lu^3+^ incorporation increases the proportion of deep traps, resulting in slow carrier release and enhanced afterglow [35].

Figure 6c shows the pulse height spectra of the ceramic samples under excitation from a 10 μCi-level ^137^Cs radioactive source (662 keV). The detailed calculation method for the light yield is provided in the Supporting Information. The scintillation photons were collected using an end-window PMT, and the spectral sensitivity of the PMT was considered in the light-yield calculation by correcting the measured pulse height according to the overlap between the sample emission spectrum and the PMT spectral response curve. With increasing Lu content, the light yield of (Lu,Gd)_3_Ga_2_Al_3_O_12_:Ce ceramics decreases from 13,837 ph/MeV at x = 0.1 to 2368 ph/MeV at x = 0.997, consistent with previous reports [35]. The relatively low measured light yield can be mainly attributed to the non-ideal optical quality and irregular geometry of the ceramic samples. As observed from the SEM images, residual pores and microstructural defects are still present in the ceramics. These defects can act as scattering and trapping centers during scintillation-light transport. This is also consistent with the relatively low optical transmittance of the samples, indicating that considerable light scattering occurs inside the ceramics. In addition, the irregular sample geometry may weaken the optical coupling between the sample and the PMT and increase interfacial reflection and light-extraction loss. Consequently, the number of photons collected by the PMT is reduced, leading to a lower measured light yield. Therefore, the light-yield values reported here are more suitable for comparing the relative scintillation performance among the present ceramic samples rather than for direct comparison with commercial single-crystal scintillators or highly optimized ceramic scintillators.

Table 2 presents the scintillation decay times, afterglow residuals, and light yield for samples with Lu concentrations ranging from x = 0.1 to x = 0.997. As shown in the table, although the light output is decreased overall with increasing Lu^3+^ doping, the decay rate is significantly accelerated. Based on these observations, we conclude that high concentrations of Lu doping significantly reduce the light output. Excessive Lu concentration causes a slow return process of Ce^3+^, leading to stronger afterglow residuals and lower light yield. These phenomena can be attributed to internal defects in the material, particularly anti-site defects. To address these issues, subsequent optimization of the preparation process could help reduce the concentration of anti-site defects.

**Figure 7:**
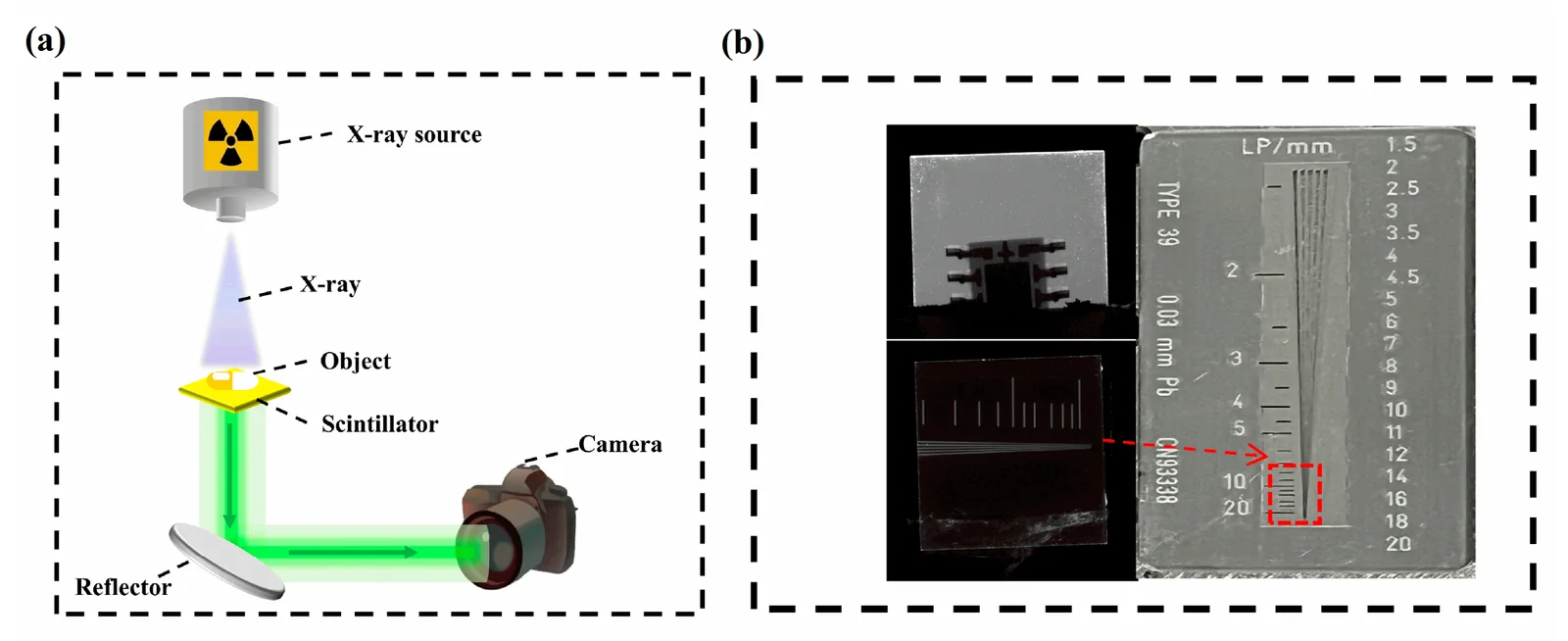
(**a**) Schematic diagram of the experimental set-up for X-ray imaging. (**b**) X-ray images of a resistor and a standard line-pair card obtained using the commercial GAGG:Ce single-crystal reference.

To evaluate the practical X-ray imaging performance of the samples, high-resolution X-ray imaging tests were conducted. For comparison, a commercial GAGG:Ce crystal was tested under the same conditions, as shown in Figure 7b. The commercial crystal provides clearer chip images and a higher spatial resolution above 20 lp mm^−1^, indicating its advantage in optical quality and imaging clarity.

**Figure 8:**
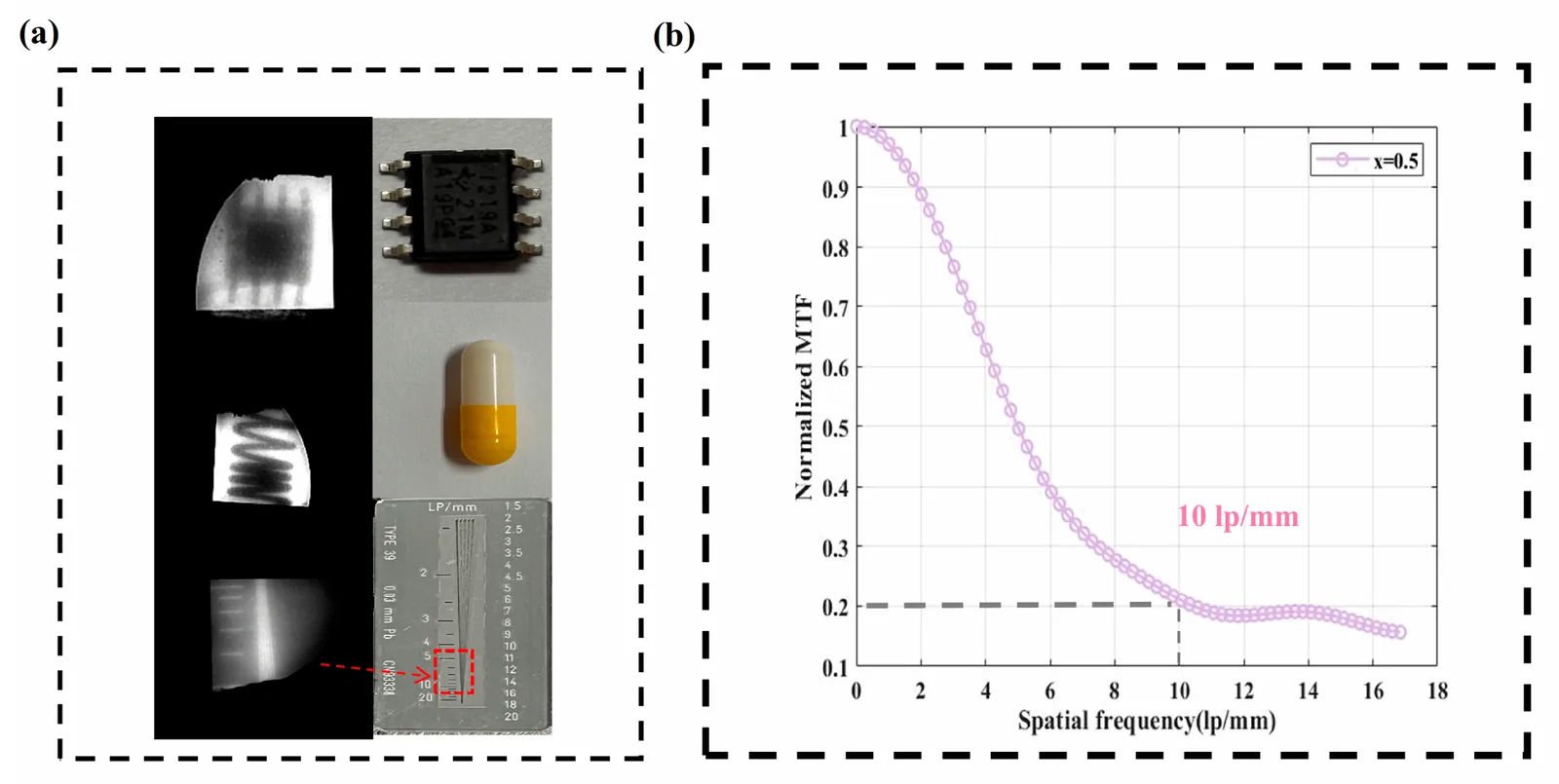
(**a**) Photographs under daylight and X-ray images of a resistor, a capsule containing a spring, and a standard line-pair card obtained using the Ce:GLAGG ceramic scintillator. (**b**) MTF curve of Ce:GLAGG ceramics.

Figure 8a shows photographs and X-ray images of a chip, a spring-containing capsule, and a standard line-pair card obtained using the x = 0.5 ceramic. The chip structure and spring outline are clearly visible, demonstrating the X-ray imaging capability of the ceramic sample. The line-pair card image further shows that the x = 0.5 ceramic achieves a spatial resolution of approximately 10 lp mm^−1^. The modulation transfer function (MTF), obtained by the edge method, was further used to quantitatively evaluate the spatial resolution of the x = 0.5 ceramic, as shown in Figure 8b. At an MTF value of 0.2, the imaging resolution is approximately 10 lp mm^−1^, comparable to the typical spatial resolution of commercial CsI:Tl scintillation crystals, suggesting its potential for X-ray imaging applications. X-ray imaging performance is jointly affected by microstructure, light yield, optical transmission efficiency, and carrier trapping behavior [44,45,46,47]. Based on the SEM and optical transmittance results, residual pores are present in the ceramic samples. These pores can act as scattering centers, causing lateral light spread and blurred image edges, thereby limiting spatial resolution [31,48]. In addition, strong afterglow increases the background gray level and may induce image lag, reducing image contrast. For the x = 0.5 ceramic, the lower afterglow intensity and weaker TL signal suggest a more favorable trap distribution, which helps suppress background noise. Meanwhile, its relatively strong XEL intensity and light output enhance imaging brightness. Therefore, the improved imaging performance of the x = 0.5 sample can be attributed to a better balance between light yield and trap suppression. These results indicate that Ce:GLAGG ceramics are promising for X-ray imaging and radiation detection, although further improvements in optical quality and afterglow suppression are still needed.

## 4. Conclusions

In this study, a series of (Gd_0.997−x_Lu_x_Ce_0.003_)_3_Ga_2_Al_3_O_12_ (x = 0.1, 0.5, 0.7 and 0.997) scintillation ceramics were prepared using the solid-state sintering method in a pure oxygen atmosphere. The effect of Lu^3+^ substitution for Gd^3+^ on the scintillation performance and the underlying mechanisms was investigated. The results show that the sample with x = 0.997 exhibits the best scintillation decay time (63 ns), while the sample with x = 0.5 displays the best afterglow performance (0.48%@100 ms), and the sample with x = 0.1 has the highest light yield (13,837 ph/MeV). Notably, (Gd_0.497_,Lu_0.5_,Ce_0.003_)Ga_2_Al_3_O_12_ ceramics achieve X-ray imaging resolution comparable to that of commercial CsI:Tl (10 lp mm^−1^). Based on these characteristics, the developed Ce:GLAGG scintillation ceramics show good potential for high-resolution imaging and high-sensitivity X-ray detection applications. However, scintillation performance still needs improvement. Future work will focus on optimizing the composition and sintering temperature to achieve higher optical quality and overall performance.

## Figures and Tables

**Table 1:** Scintillation properties of representative commercial X-ray scintillators.

Material	Density (g/cm^3^)	Light Yield (ph/MeV)	Decay Time (ns)
CsI:Tl	4.51	61,000	630
GOS	7.34	45,000	600
CdWO_4_	7.9	13,000	14,000
LYSO	7.1	32,000	41
GAGG	6.63	50,000	88

**Table 2:** Scintillation properties of Ce:GLAGG ceramics with different Lu^3+^ doping concentrations.

Samples	Average Decay Time (ns)	1st Decay Time (ns)	2nd Decay Time (ns)	Afterglow @100 ms (%)	Light Yield (ph/MeV)
x = 0.1	317	126	484	0.99	13,837
x = 0.5	265	63	393	0.48	9972
x = 0.7	278	73	468	0.57	13,089
x = 0.997	63	48	352	5.8	2368

## Data Availability

The original contributions presented in this study are included in the article/Supplementary Materials. Further inquiries can be directed to the corresponding authors.

## Supplementary Materials

The following supporting information can be downloaded at: https://www.mdpi.com/article/10.3390/ma19173574/s1.
