# Supplementary material for: Lu3+ Substituted Gd3Ga2Al3O12:Ce Ceramics for Improved X-Ray Imaging
Source: Materials (Basel). 2026 Aug 23;19(17):3574. doi: 10.3390/ma19173574 (PMC13566179; doi:10.3390/ma19173574)
Supplement: Supplementary file 1 [file materials-19-03574-s001.zip › materials-4424447-supplementary.pdf]

Supplementary Material

Lu^{3+} Substituted $Gd_3Ga_2Al_3O_{12}$:Ce Ceramics for Improved X-Ray Imaging

Yuetong Zhen [1], Hui Lin [1,*], Yang Tang [1], Junwei Zhang [1], Yuchong Ding [2], Qiang Wang [2], Dawei Zhang [1] and Jianren Xu [1,*]

[1] *Engineering Research Center of Optical Instrument and System, Ministry of Education and Shanghai Key Lab of Modern Optical System, University of Shanghai for Science and Technology, No. 516 Jungong Road, Shanghai 200093, China*

[2] *Research & Development Center of Materials and Equipment, China Electronics Technology Group Corporation No. 26 Research Institute, Chongqing 400060, China*

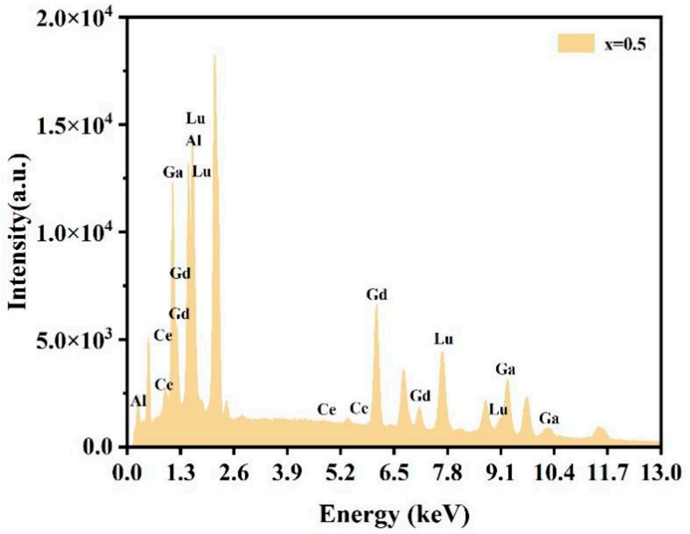

Figure S1 Point EDS spectrum of $(Gd_{0.497}Lu_{0.5}Ce_{0.003})_3Ga_2Al_3O_{12}$ ceramic.

Table S1 Semi-quantitative point EDS results of the x = 0.5 ceramic.

Element	Weight percentage (wt%)	Atomic percentage (at%)	Net intensity
Al K	10.1	35.0	484
Ga K	18.1	24.4	393
Ce L	0.7	0.4	468

Gd L	33.2	19.8	352
Lu L	38.0	20.4	337.0

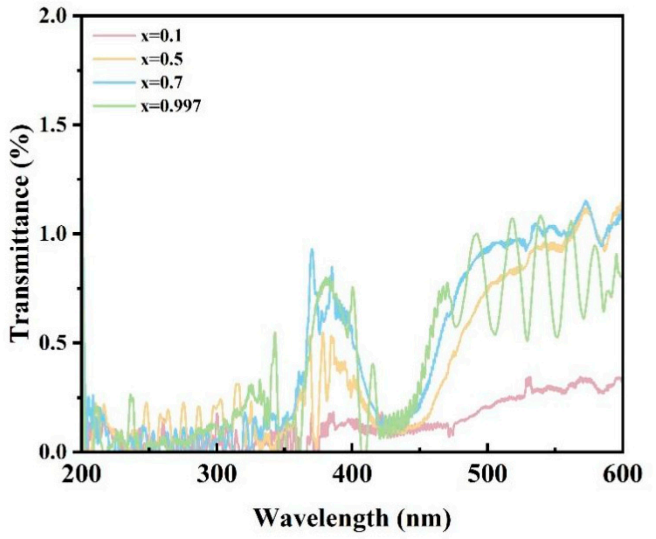

Figure S2 Transmission spectra of Ce:GLAGG transparent ceramics.

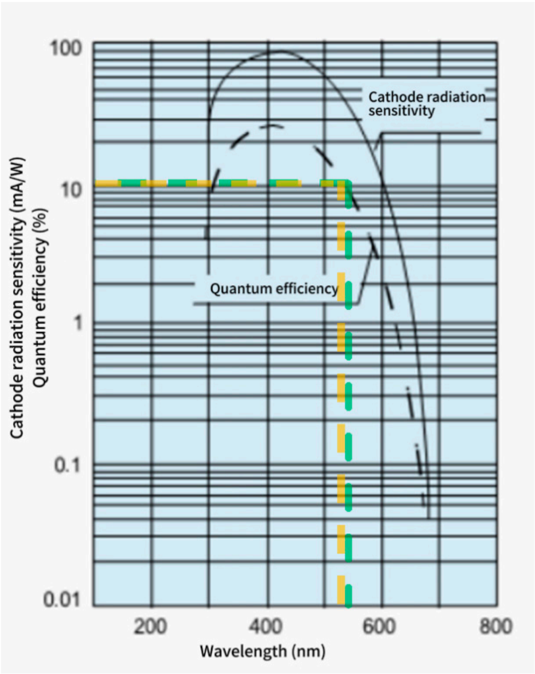

Figure S3 The quantum efficiency curve of the Hamamatsu CR105 PMT.

The detailed calculation of light yield is shown below:

The commercial GAGG:Ce scintillator with a light yield of 46,000 ph/MeV was used as a reference to calibrate the light yield of the samples.

$$LY_M = \frac{LO_M}{LO_S} LY_S \eta \qquad \text{S1}$$

$$\eta = \frac{\eta_{GAGG}}{\eta_{GLAGG}} \qquad \text{S2}$$

LY_M is the light yield of the sample to be measured, LO_M is the channel number of the samples, LO_S is the channel number of the reference sample, LY_S is the light yield of the reference sample, and η is the quantum efficiency. The quantum efficiency is shown in Figure 1, from which it can be observed that both GAGG and GLAGG exhibit quantum efficiencies of approximately 10%.
